# Effect of the Application of Sunflower Biochar and Leafy Trees Biochar on Soil Hydrological Properties of Fallow Soils and under Soybean Cultivation

**DOI:** 10.3390/ma16041737

**Published:** 2023-02-20

**Authors:** Urszula Sadowska, Tomasz Zaleski, Maciej Kuboń, Agnieszka Latawiec, Agnieszka Klimek-Kopyra, Jakub Sikora, Maciej Gliniak, Rafał Kobyłecki, Robert Zarzycki

**Affiliations:** 1 Department of Machinery Exploitation, Ergonomics and Production Processes, Faculty of Production Engineering and Energetics, University of Agriculture in Krakow, Balicka 116 B, 30-149 Krakow, Poland; 2 Department of Soil Science and Agrophysics, University of Agriculture in Krakow, Al. Mickiewicza 21, 30-120 Krakow, Poland; 3 Department of Production Engineering, Logistics and Applied Computer Science, Faculty of Production and Power Engineering, University of Agriculture in Krakow, Al. Mickiewicza 21, 30-120 Krakow, Poland; 4 Department of Geography and Environment—Rio Conservation and Sustainability Science Centre, Pontifical Catholic University of Rio de Janeiro, R. Marquês de São Vicente, 225, Gávea, Rio de Janeiro 22451-000, Brazil; 5 International Institute for Sustainability, R. Dona Castorina 124, Jardim Botânico, Rio de Janeiro 22460-320, Brazil; 6 Faculty of Mechanical Engineering, Opole University of Technology, Mikołajczyka 5, 45-271 Opole, Poland; 7 School of Environmental Sciences, University of East Anglia, Norwich Research Park, Norwich NR4 7TJ, UK; 8 Department of Agroecology and Plant Production, Faculty of Agriculture and Economy, University of Agriculture in Krakow, Mickiewicza Av. 21, 31-120 Krakow, Poland; 9 Department of Bioprocess Engineering, Faculty of Production and Power Engineering, Power Engineering and Automation, University of Agriculture in Krakow, Balicka 116B, 30-149 Krakow, Poland; 10 Department of Advanced Energy Technologies, Czestochowa University of Technology, Dąbrowskiego 69, 42-201 Czestochowa, Poland

**Keywords:** soil amendment, biochar, retention, soybean porosity, total surface area

## Abstract

Soils enriched with biochar are recommended as a cultivation grounds, especially in case they contain significant amount of sand. However, the interactions between biochar and plants, as well as the influence of the biochar on water retention, cultivation and air properties of soils, are still not obvious. The present study aimed to determine the impact of various biochar doses on soils used for soya cultivation, in comparison to soils maintained as black fallow soil, on their water retention and productivity, for the period of two years. Sunflower husk biochar (BC1) and biochar of leafy trees (BC2), in doses of 0, 40, 60, 80 t·ha^−1^, were used for field experiments. The water retention was investigated with porous boards in pressure chambers by a drying method. No differences in the hydrological properties of the soils that were differently managed (black fallow soil, crop) were observed following biochar application. Addition of BC1, in the amounts of 40, 60, and 80 t·ha^−1^, caused an increase in the plant available water capacity (AWC) by 15.3%, 18.7%, and 13.3%, respectively, whereas the field capacity (FC) increased by 7.4%, 9.4%, and 8.6% for soils without biochar. Application of BC2 analogously resulted in higher AWC, by 8.97, 17.2%, and 33.1%, respectively, and higher FC by 3.75, 7.5%, and 18.3%, respectively. Increasing the doses of BC1 and BC2, both on black fallow soils and soils enriched with soya, caused a rise in total porosity (TP) and drainage porosity (DP), and a decrease in soil bulk density (SBD). Biochar with a higher total area and higher porosity (BC1) applied to soils with soya cultivation resulted in lower reductions in AW and FC than BC2 in the second year of investigation.

## 1. Introduction

Research studies on biochar (biocarbon), and related issues such as its production by pyrolysis, its properties, and the wide scope of its possible applications, have become one of the main research subjects in recent years. However, deficiencies in knowledge and inconsistencies in the obtained results are still reported. Adding biochar to soil is commonly recognized as an efficient method of carbon sequestration and improvement of soil fertility. A considerable increase of biochar use in agriculture has been reported since 2015 [1]. However, the effects of biochar use in agriculture vary and depend on many factors: such aus the plant type, the biochar properties and the dose of application, and some soil properties and environmental conditions [2]. 

The biochar, after its introduction to soil, may change the parameters of soil water retention [3] and the availability of nutrients [4]. Many studies proved that, according to the initial properties of the substrates and the parameters of pyrolysis, the biochars have different properties, with respect to their carbon and ash content, pH, surface area, adsorption of nutrients, porosity, and water retention ability [5,6,7,8,9]. Raw materials have characteristic physico-chemical properties due to the differences in their elemental compositions and structures, and do not react uniformly to specific pyrolysis conditions. In their meta-analysis, Hassan et al. [9] have shown that the criteria of selection of raw materials and pyrolysis conditions for tailoring the biochars with regard to their specific sorption properties are insufficiently recognized and often the processed biochars maintain some properties of the original feedstock [10]. According to Zhao et al. [11], the total participation of organic carbon, bound carbon, and mineral components content should be included as the parameters of the raw material may significantly affect the properties of the produced biochar. The quoted authors claim that the potential total C sequestration is mainly determined by the type of the feedstock.

The physico-chemical properties of the biochars may vary over a wide range [12]. Short-term changes in the physical and hydrological properties of soil enriched with biochar were observed in many laboratories. Razzaghi et al. [3] and Edeh et al. [1] emphasize that biochar particularly favorably influences weak, sandy soils, where hydrological properties have been noted. In some case, the application of biochar may increase water retention and also the amount of water available for plants [1]. In a few studies it has been shown that the addition of 10 t·ha^−1^ of biochar did not affect the AWC, but higher doses considerably increased the amount of available water [13,14]. After the application to soil the properties of the biochar, such as particles size, internal porosity, and water repellence may change over time affecting the total water retention [15]. 

Soya (Glycine hispida) belongs to leguminous plants and is one of the most valuable plants in the world, providing the highest yield of high-quality protein from a hectare FAO (2010) [16], and because of that, it is extensively used [17]. In agriculture, soybean improves the biodiversity of the crop structure, and due to the symbiosis with *Bradyrhizobium japonicum* bacteria it can be cultivated without nitrogen fertilization, thus contributing to the reduction in the carbon footprint. So far, thecultivation of soybean has been concentrated mainly in the USA, Brazil, Argentina, and China [18] and EU countries, including Poland are significant importers of soya and soya meal [19]. In Poland and other EU countries, actions are taken to stimulate the development of the market of native protein crops [20,21]. There is an expectation that in the coming years theproduction of soybean will increase more than other crops due to the enlargement of the production area. Studies by Arata et al. (2020) [22] indicated that the geographical region is a key factor influencing the yield variability. Abiotic limitations of the seed yield are related to extreme values of nutrients, temperature, and humidity [23]. Starkel and Kundzewicz (2008) [24] reportedthat, due to a statistically-proven increase in the average air temperature in the last 12 years and thus the warming of the climate in Poland, thermophilic plants, including soya, currently behave more favorably with regards to their development and production yield. However, especially on sandy soils with low retention capacity, the unevenness of precipitation, and consequently periodic droughts, might be a problem. Therefore, there is a search for various solutions to improve the water retention of such soils. In an extensive meta-analysis Blanco-Canqui [25] concluded that biochar addition improved the physical soil environment and changed its total porosity, as well as the distribution of pore sizes, water retention ability, and the retention characteristics. Numerous studies have shown the potential of the biochar as a non-structural BMP for improving soil quality and sustainability and alleviating its erodibility. As soil component the biochar also helps to prevent the decrease in SOM (soil organic matter) and thus soil degradation, as well as the leaching of salts or nitrogen-compounds into the water table that is the result of intensive fertilization and a serious problem of modern farming. The biochar may also reduce the release of CH_4_ and N_2_O (both greenhouse gases) from natural decay processes in soils, particularly in wetlands, that are significant methane producers on the planet. It may also be an appropriate sorbent for the removal of certain contaminants from degraded or low-quality soils and is considered a low cost ’green’ alternative to commercial activated carbon, also making the biochar an appropriate tool for long-term sequestration of atmospheric carbon dioxide.. Accordingly, the production of biochar and its sequestration in soil seems to be an efficient and easy way to counteract climate changes and a useful tool for the storage of carbon outside the atmosphere [26,27,28].

Generally, the effect of biochar addition on soil properties and plant growth and yield is more obvious for less fertile soils compared to more fertile ones, particularly those with a suitable moisture content [1,29]. In agronomy, the application of biochar is still a subject of research, since the complexity of interactions between plants, soil, and biochar may be very substantial and may determine the obtained agronomic effects [30,31]. Moreover, there are discrepancies between the results of pot experiments compared to the field ones and quite often only the pot experiment data are taken into consideration [32]. Edeh et al. [1] noted for instance that the majority of current studies associated with the agronomic use of biochar were carried out in laboratories, and in a considerable number of cases the investigations and measurements were conducted right after the mixing of the biochar and soil. Thus, long-term field studies are lacking particularly those that could better reflect the soil-biochar interactions in order to assess and determine the most optimal biochar dosis [1,25]. 

The objective of this paper is thus focused on the determination of the time-effects of biochar application on water and air/hydrophysical properties of fallow soils and the soils cultivated with soya. Various additive doses and two types of biochar were investigated in the current study. 

## 2. Materials and Methods

### 2.1. Biochar Production

**Figure 1:**
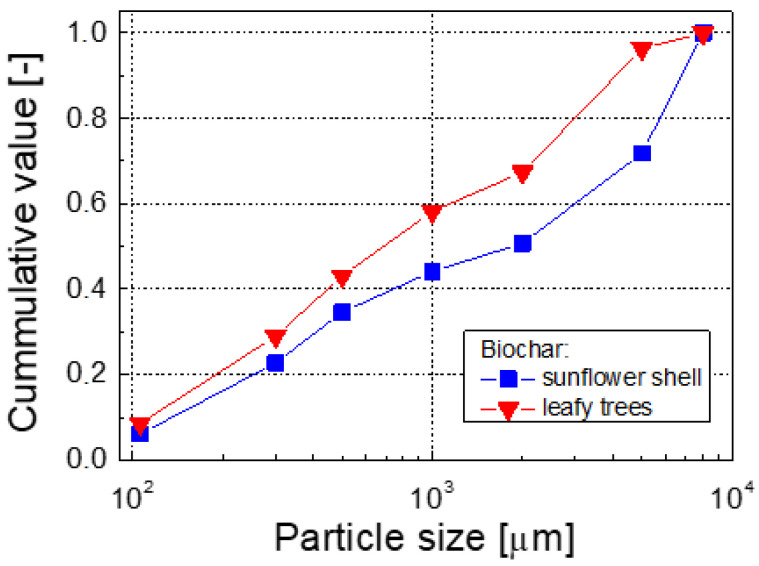
Granulation (particle size distribution) of the biochars produced.

Biochar (BC) was produced by thermal treatment (thermolysis) of the biomass feedstock in a pilot auger-type horizontal reactor (i.d. 0.1 m), designed by the authors and manufactured by a small company in Poland.. The reactor was electrically heated and the heated section was 3 m long. The biomass was continuously fed and discharged from the reactor by two independent screw transporters also manufactured by a small Polish company. The temperature was maintained at 440–480 °C throughout the process. Two raw materials were chosen for the biochar production, sunflower husk pellets and wood pellets (leafy trees). The biochar produced from these feedstocks was named BC1 and BC2, respectively. The chemical compositions and basic physical properties of the biomasses and the biochar are shown in Table 1 and Table 2 and Figure 1. As for the feedstocks, both biomasses are mainly composed of volatile matter (VM) and are characterized by low (<2%) ash content. The C, H, and N contents are typical for those type of materials, as well as their energy content (HHV and LHV) [33,34]. The data shown in Table 1 also indicate that, due to thermal treatment of the feedstock, most volatiles had ‘evaporated’, and compared to the raw materials, the VM content in the biochar is significantly lower (13.8% and 8.6% for BC1 and BC2, respectively). Due to the loss of the VM gases, the carbon and ash contents in the solid residues (the biochar) had also significantly increased (cf. the corresponding values in Table 1). 

### 2.2. Field Experiment

The field experiment was conducted during the years 2019–2020, on the experimental field of the University of Agriculture in Krakow (50°04′ N, 19°51′ E, 211 m MSL, slope 2°), on light silty soil (sand (56.7%), silt (32%), and clay (10.4%), with a gravel fraction (0.9%)). The soil was characterized as Calcaric/Dolomitic Leptosols (Ochric) [35]. In spring 2019, two types of biochar (BC1 and BC2) were applied manually. The biochar was mixed into the soil with a manual rotary tiller in a layer to a depth of 20 cm. 

The biochar doses were established based on the results of the initial experiment carried out in 2018, and for each of the used biochars (BC1 and BC2) were 0, 40, 60, and 80 t·ha^−1^. The tests were performed both on samples of soils collected from the fields where previously soya was cultivated, as well as from fields maintained as black fallow land. Each variant of the experiment was carried out in three iterations. On all facilities, uniform fertilization was applied (30 kg N, 70 kg P_2_O_5_, 100 kg K_2_O). During vegetation, manual weeding was performed several times. Soils were not loosened as a result of the treatment that was carried out. No chemical protection substances were used in the experiment. The soya was sown in a standard dose of (80 seeds·m^−2^). Before sowing, the seeds were inoculated every year with *Bradryzobium japonicum* bacteria. The soya yield was assessed when fully matured based on the yield structure. Every year after the soya harvest (September), soil samples were collected for tests from the layer of 5–15 cm depth, to which the biocarbon was introduced.

The samples for tests were collected from facilities with a specific BC1 (0, 40, 60, and 80 t·ha^−1^) and BC2 (0, 40, 60, and 80 t·ha^−1^) content in the fallow soil and in the soil covered with plants. From the depth of 5–15 cm, samples in an untouched distribution were collected to cylinders with volumes of 100 cm^3^, for tests on their hydrophysical properties. The tests were performed both on samples of soils collected from the fields where previously soya was cultivated, as well as from fields maintained as fallow land. The research was carried out in three iterations for each combination.

### 2.3. Laboratory Experiment

The soil moisture characteristics were determined in pressure chambers with ceramic plates, according to the method described by Richards [36]. Undisturbed soil samples were collected using metal cylinders of 100 cm^3^ capacity. 

For the soil water retention measurements of experimental facilities, a standard laboratory procedure was used. The water retention parameters were determined with a ceramic porous plate in pressure chambers (made by Eijkelkamp, Giesbeek, The Netherlands), according to the method described by Richards [36]. In cylinders with a volume of 100 cm^3^, with an untouched structure, within the range of potentials from pF = 1.0 to pF = 3.0, and in soil samples with an undisturbed structure placed in rings of 1 cm height, values for the potency of pF = 4.2 were calculated. pF is an international unit of soil water potential introduced by Schofield in 1935, which expresses the negative pressure of water on a logarithmic scale.

The soil water retention curves (pF curve) were expressed by the following equation and were modelled used the RETC 6.02 software [37],

(1) $$\theta \left(h\right)=\theta_{r}+\frac{\theta_{c}-\theta_{r}}{{\left(1+{\left|\alpha \cdot h\right|}^{n}\right)}^{m}}$$

where, θ is the water content in the soil (m^3^·m^−3^), θ_r_ is the residual water content in the soil (m^3^·m^−3^), θ_c_ is the water content in the soil in saturation state (m^3^·m^−3^), α is the empirical constant of a curve, n is the empirical constant of the curve shape, m = 1 − 1/n, and h is the soil water potential (cm).

In all the collected soil samples, the following water and soil constants and physical parameters were determined:

1. Field capacity (FC), water content at field water volume (pF = 2.0).
2. Permanent wilting point (PWP), a limit of water unavailability for plants’ moisture corresponding to pF = 4.2 was assumed.
3. Available water capacity for plants (AWC):

(2) AWC = FC−PWP

4. Soil bulk density of dry soil, SBD (ρc) in cylinders of volume 100 cm^3^ [38].
5. Total porosity (TP) was calculated based on the value of the constant phase of soil ρs and soil bulk density ρc.

(3) TP =(ρs−ρc)/ρs

6. Drainage porosity (DP), as a difference between the general porosity TP and field capacity FC.

### 2.4. Statistical Analysis

The tests results were analyzed in terms of variance with the use of the STATISTICA 12 software. The significance of the obtained differences were verified with Tukey’s test at the level of significance of *p* ≤ 0.05.

The obtained results were analyzed using two approaches:

1. The impact of BC1 and BC2 was subjected to a separate analysis.
2. A simultaneous analysis was made on the impact of BC1 and BC2 on fallow soils and the soils where soya was cultivated.

## 3. Results

### 3.1. Meteorological Conditions 

**Figure 2:**
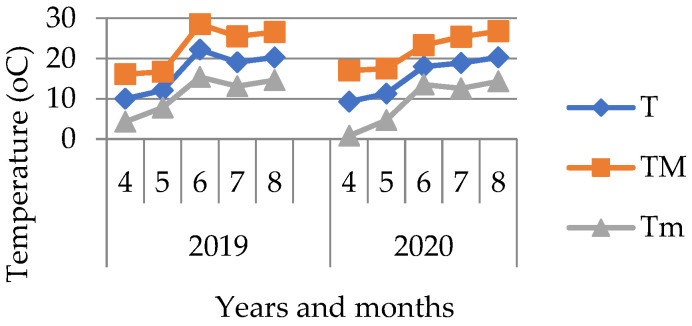
Air temperature distribution during tests. Average (T), maximum (TM), and minimum (Tm) temperatures in particular soya vegetation months in the years of the tests.

**Figure 3:**
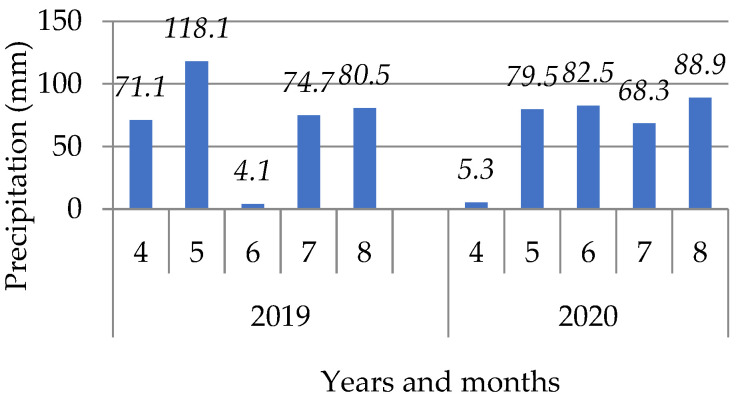
Total monthly sum of precipitation in particular months of soya growing in the years of the research.

The data were obtained from a meteorological station Krakow, Balice (50°08′ N, 19°78′ E, 237 m MSL). In the investigated growing seasons, the course of the weather was similar, however, it may be noticed that 2019 was slightly warmer in comparison to 2020, and in June, high temperatures were observed (Figure 2). June 2019 has the lowest average precipitation, only 4.1 mm (Figure 3). A high temperature and low amount of precipitation favors evaporation and thus may certainly cause soil water deficiencies.

Based on the presented average values of precipitation in the particular months of the soya growing season, a more even distribution of precipitation in 2020 is noticeable. This year, April had the smallest amount of precipitation. In 2019, the lowest precipitation was reported in June.

### 3.2. Impact of Biochar Used on Selected Hydrological and Physical Soil Properties

**Figure 4:**
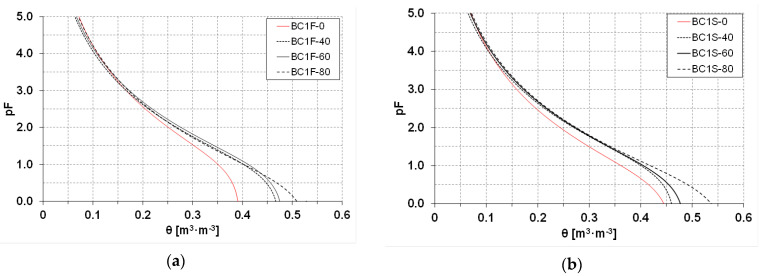
Average (2019–2020) soil water retention curves (SWRC) with BC1 additions within the range of 0–80 tons per hectare, (**a**) on fallow land fields BC1F, (**b**) on fields with soya BC1S.

**Figure 5:**
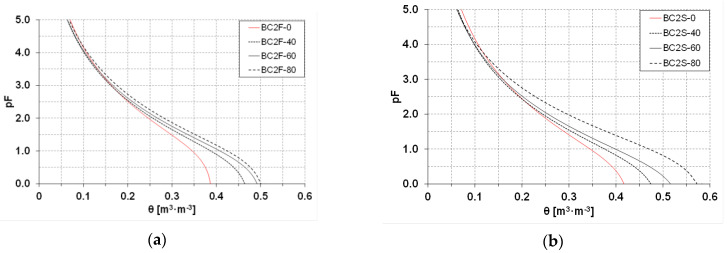
Average (2019–2020) soil water retention curves (SWRC) with BC2 additions within the range of 0–80 tons per hectare, (**a**) on fallow land fields BC2F, (**b**) on fields with soya BC2S.

The obtained curves of soil retention capability (SWRC) are presented in Figure 4 and Figure 5 as average values for the period 2019–2020.

A graphical presentation of the water relations of the investigated soils (Figure 4 and Figure 5) shows curves with a similar shape. A slightly flatter curve of soil retention, in the case of the soil covered with plants, regardless of which biochar is used, is seen. Moreover, for soils used for soya cultivation where BC2 was applied, at the same pressure values, in comparison to analogous BC1 doses, the water content in the soil is higher. 

In Table 3 and Table 4, the average values of water and soil constants for the period 2019–2020, and selected physical properties of the investigated soils, are presented in relation to the dose and type of biochar and the manner of management.

Based on the performed statistical analysis of the obtained two-year test results, a significant impact of the applied biochar on the soil retention properties was shown. In comparison to facilities where no addition was used, BC1 improved the soil retention properties. A higher field capacity and available water capacity occurred in the cases of soils where biochar was applied, regardless of the applied dose. The increase in biochar BC1 participation caused the increase in the general porosity and drainage porosity values and a reduction in the bulk density of dry soil. In the case of soils enriched with BC1, no difference in the values of the investigated parameters was reported due to the manner of cultivation. In 2019, in comparison to 2020, higher values of general porosity, drainage porosity, field capacity and available water capacity, and lower values of bulk density of the dry soil, were obtained.

With an increasing dosage of BC2, increases in the general porosity and drainage porosity, and available water capacity were reported, and a reduction in the bulk density of dry soil was reported. Values of the field capacity differed only significantly between the control soil and the soil enriched with this component, regardless of the applied dose. An increase in the general porosity, drainage porosity, and bulk density of the dry soil of facilities where soya was cultivated, in comparison to fallow land, was observed.

Also, after the application of BC2, differences between the two years of study were reported. Comparing 2019 to 2020, higher values of the general porosity, field capacity, permanent wilting point, and utility water content, and a lower value of bulk density of the dry soil, were reported.

**Figure 6:**
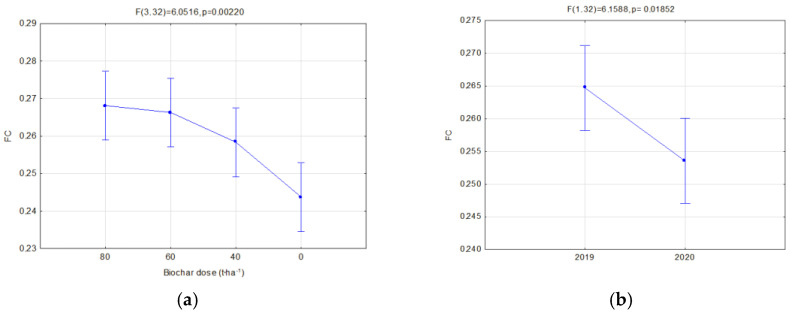
Field capacity (cm^3^·cm^−3^) of fallow soils in relation to the dose applied (**a**) and year of research (**b**).

**Figure 7:**
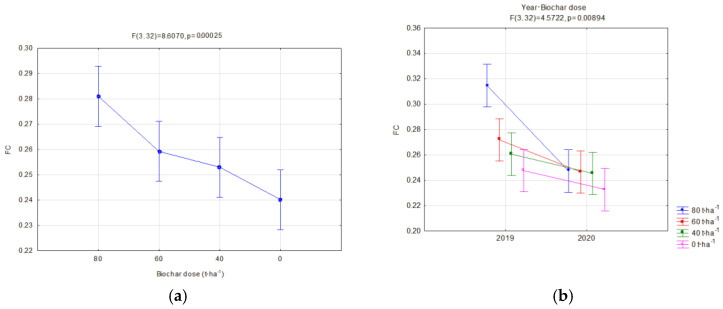
Field capacity (cm^3^·cm^−3^) of soils with soya in relation to the dose applied (**a**) and interaction of the biochar dose amount and year of research (**b**).

**Figure 8:**
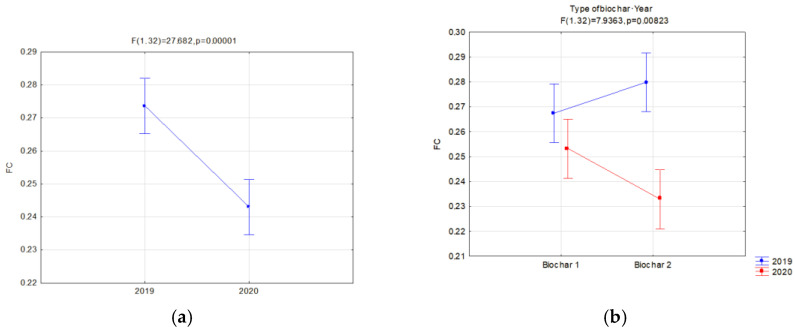
Field capacity (cm^3^·cm^−3^) of soils with soya in relation to the year of research (**a**) and interaction of biochar type and year of research (**b**).

**Figure 9:**
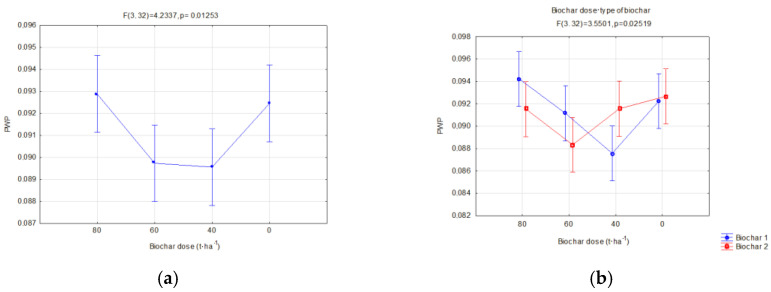
Permanent wilting point (cm^3^·cm^−3^) of fallow soils in relation to the applied biochar dose (**a**) and interaction of a dose and biochar type (**b**).

**Figure 10:**
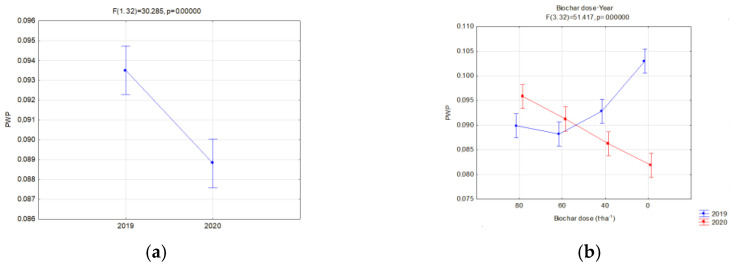
Permanent wilting point (cm^3^·cm^−3^) of fallow soils in relation to the year of research (**a**) and interaction of a dose and year of research (**b**).

**Figure 11:**
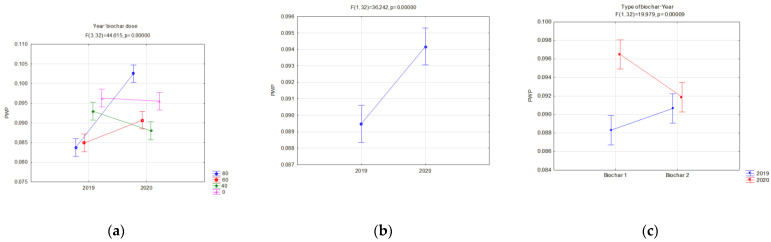

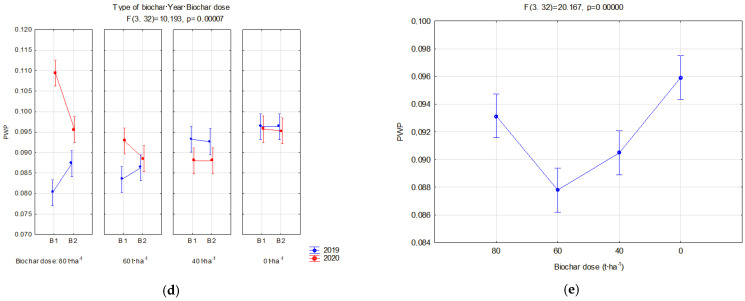
Permanent wilting point (cm^3^·cm^−3^) of soils with soya, significant relations and all interactions; interaction of the dose amount and year of research (**a**), in relation to the year of research (**b**), interaction of biochar type and the year of research (**c**), interaction of the dose amount, biochar type and year of research (**d**) and in relation to the dose applied (**e**).

**Figure 12:**
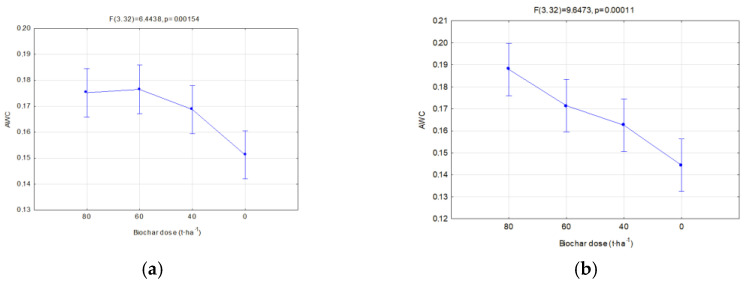
AWC (cm^3^·cm^−3^) for fallow soils (significant only dose) (**a**) and for soils covered with plants in relation to the applied dose of biochar (**b**).

**Figure 13:**
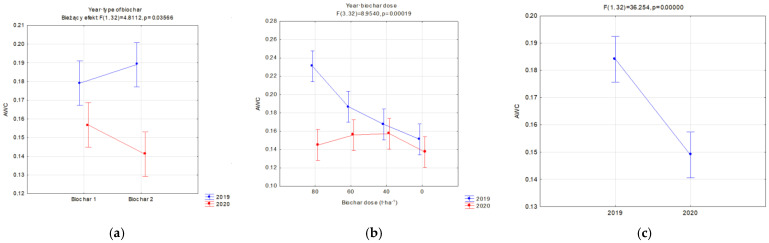
AWC (cm^3^·cm^−3^) for soils covered with plants, significant relations; in relation to the applied dose of biochar (**a**), dose interaction and year of research (**b**) and in relation to the year of research (**c**).

Moreover, the soils’ reaction to the impact of application of similar doses of two types of biochar was compared. A separate analysis was carried out in relation to the manner of use (fallow land and covered by soya cultivation) for two types of biochar simultaneously in order to find the most favorable variants of retention and physical properties of the investigated soils. Significant relations concerning the hydraulic properties are presented in Figure 6, Figure 7, Figure 8, Figure 9, Figure 10, Figure 11, Figure 12 and Figure 13. 

The field capacity (pF 2.0) significantly depended on the applied biochar dose. Addition of biochar increased the field capacity. A dose of 80 t·ha^−1^ on fallow lands caused an average increase in FC of 9.99% in comparison to soils without biochar (Figure 6a). Similarly, a dose of 60 t·ha^−1^ caused an increase of 9.24%. Whereas, in soils with soya, a biochar dose of 80 t·ha^−1^ caused an FC increase of 16.99% (Figure 7a). A dose of 60 t·ha^−1^ on soya soils caused an average increase in FC of 7.9% in comparison to soils without biochar. The biochar type did not affect the FC on fallow lands. In soils with soya, the FC was affected by an interaction of the biochar type and the year of research (Figure 8b). On these soils, also a higher reduction in FC for BC2 within one year after its application was observed (Figure 8a). In the second year of research, on fallow lands, a lower value of FC was obtained, on average by 4.23% (Figure 6b), whereas, for soils covered with soya, the decrease was 10.17% (Figure 8a) in comparison to the previous year. In facilities where soya was cultivated with the biochar dose of 80 t·ha^−1^, the highest reduction in FC in the second year of research was reported, by as much as 21.74% (Figure 7b).

Similarly, the moisture was compared at which soil retains water with a power higher than the roots suction power, called the permanent wilting water content (PWP).

Biochar doses cause changes in the permanent wilting water content. The lowest PWP values on fallow soils were observed for doses of 60 and 40 t·ha^−1^.

Biochar with a lower total surface area and lower porosity (BC2), in soils with soya cultivation, gave an effect in the form of higher PWP reductions in the second year of research.

The interaction of the applied doses of biochar and the year of use influenced the PWP of soils with soya (Figure 11). High doses of biochar (80 and 60 t·ha^−1^) used in the year of application caused a reduction in PWP (Figure 11). The lowest value was obtained for BC1 (0.08 cm^3^·cm^−3^) at a dose of 80 t·ha^−1^. In the following research year, BC1 applied at the same dose gave the highest values of PWP (0.109 cm^3^·cm^−3^). The lowest PWP values in both years of research were obtained for the dose of 60 t·ha^−1^.

Changes in the FC and PWP were reflected in the AWC. The available water capacity (AWC) for plants is one of the basic indices which valorizes the agricultural properties of soils. On fallow soils, differences between the analyzed biochars were insignificant, whereas a favorable impact of the applied dose, expressed with the increase in the AWC, value was visible (Figure 12a). Similar relations occurred in soils with soya (Figure 12b). In this case, however, differences in the AWC values for particular biochars and the year of research, occurred. Interactions of the type of applied biochar and year of experiment influenced the AWC. In the application year of biochar, lower AWC values, in comparison to BC2, were reported on fields with BC1. This may be related to the biochar structure (Table 2). However, in the following year of research, the reduction in the AWC value on facilities with BC1 was lower in comparison to those with BC2. Moreover, an interaction of the applied biochar dose and years was proved (Figure 13b). The highest AWC value for soils sown with soya was obtained in 2019 for BC2, and was on average 0.189 cm^3^·cm^−3^. A dose of 80 t·ha^−1^ on these soils in 2019 caused the AWC to increase to 0.23 cm^3^·cm^−3^. Differences in the AWC levels between the years of research were reported. Regardless of the biochar dose and type, a considerable reduction in the AW levels in 2020 was reported in comparison to the biochar implementation, on average by 19.1 % (Figure 13c). The highest reduction in the AWC level, on average by ca. 37%, occurred at the biochar dose of 80 t·ha^−1^ (Figure 13b).

Considering the productive functions of agricultural soil, and water retention in the soil, the most important indicator is the AWC, because it shows the amount of water that plants can use. On vegetated soils in the first year of the study, BC2 produced higher AWC levels than BC1. However, in the second year of the experiment, there was a sharp decrease in the AWC for BC2, especially at the highest dose of 80 t·ha^−1^. Hence, in a further perspective (two years of use), BC1 is recommended at a dose of 60 or 40 t·ha^−1^.

**Figure 14:**
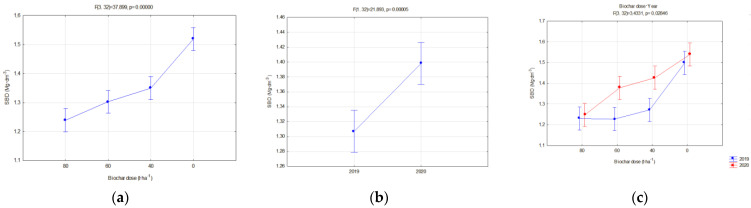
Soil bulk density for fallow soils in relation to the biochar dose (**a**), years of application (**b**) and dose interaction and the year of research (**c**).

**Figure 15:**
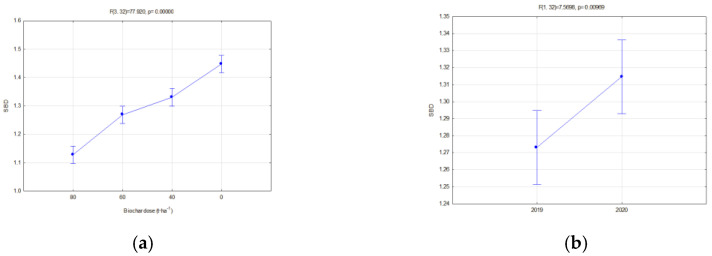
Soil bulk density for soils taken up by soya cultivation in relation to the biochar dose (**a**) and year of application (**b**).

**Figure 16:**
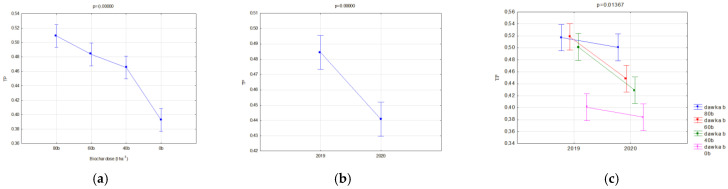
Total porosity of fallow soils in relation to the dose (**a**) and year of research (**b**) and dose interaction and the year of research (**c**).

**Figure 17:**
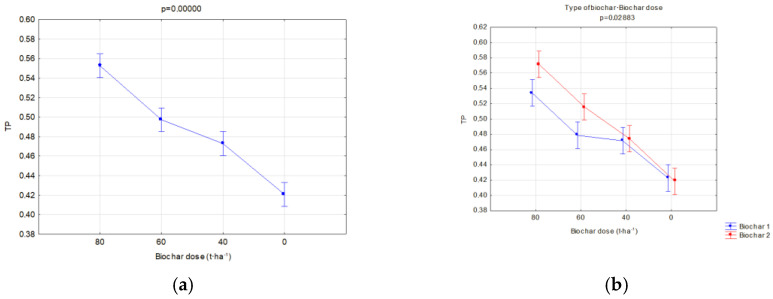
Total porosity of soils covered with soya in relation to dose (**a**) and interaction of a dose and biochar type (**b**).

**Figure 18:**
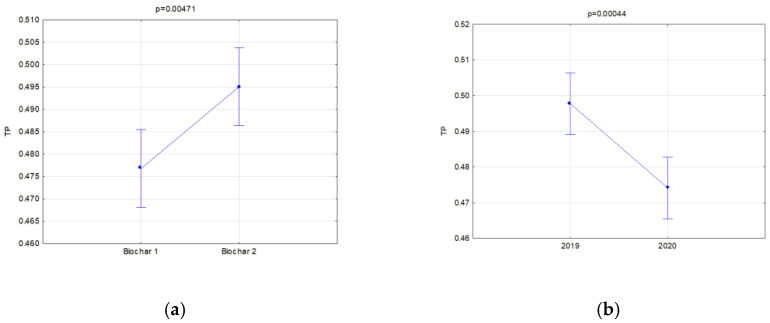
Total porosity of soils covered with soya cultivation in relation to biochar type (**a**) and the year of research (**b**).

**Figure 19:**
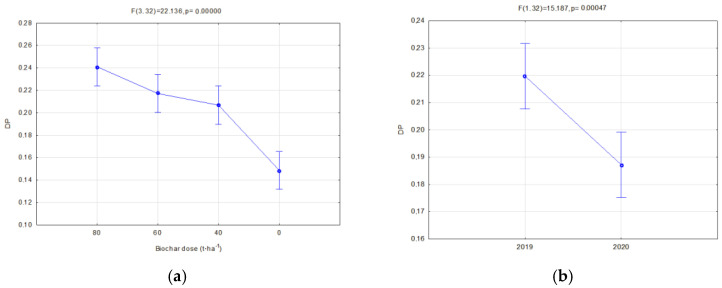
Drainage porosity of fallow soils in relation to the applied biochar dose (**a**) and the year of research (**b**).

**Figure 20:**
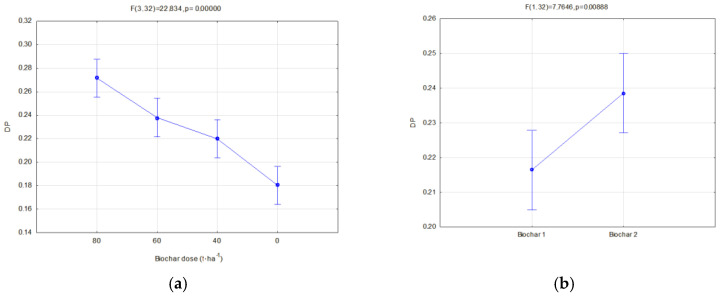
Drainage porosity of soils covered with soya crop in relation to the applied dose (**a**) and type of biochar (**b**).

The investigated physical parameters of soils from particular experiment facilities were compared. The results of these analyses are presented in Figure 14, Figure 15, Figure 16, Figure 17, Figure 18, Figure 19 and Figure 20.

Reduction in the soil bulk density under the impact of increasing biochar doses, regardless of the type and manner of soil management (fallow land/crop), was achieved. Both on fallow lands and those planted with soya crops, an increase in the soil bulk density in the second year of research was reported, by 6.96 and 3.26%, respectively, regardless of the applied dose.

In all fields, it was concluded that when the biochar dose increased, the bulk density and general porosity decreased (Figure 16a and Figure 17a). In the second year of research, a reduction occurred in the porosity in the fallow soils, on average from 0.484 to 0.440 (Figure 16b), whereas in soils with soya this was from 0.497 to 0.474 (Figure 18b). The reduction in the porosity of the fallow soils strongly depended on the biochar dose (Figure 16c). The scale of this effect depended on the applied biochar dose. For soils where 40 t·ha^−1^ and 60 t·ha^−1^ of biochar was added, the general porosity was reduced by 14.38 and 13.46%, respectively. 

In soils where soya was cultivated, BC2 increased the soil porosity much more than BC1 (Figure 18a), but significant differences occurred after application of the highest biochar doses of 60 and 80 t·ha^−1^ (Figure 17b).

Biochar application on soil caused an increase in the drainage porosity. In soils of all facilities, drainage porosities increased along with increasing biochar doses (Figure 19a and Figure 20a). In fallow soils, the drainage porosity in 2020 was reduced by 14.79% in comparison to the previous year (Figure 19b). Such a relation did not occur in the soils sown with soya. In soils where soya was cultivated, BC2 biochar caused a higher increase in the drainage porosity than BC1, on average by 10.25% (Figure 20b).

The applied biochar dose was strictly correlated with physical properties of both fallow soils and soya-cultivated ones (Table 5 and Table 6). On both types of soils, also a significant positive correlation of the biochar dose with the FC and AWC levels was reported. The analyzed hydrological and physical parameters of the soils depended on the used biochar type.

### 3.3. Soya Yield

**Figure 21:**
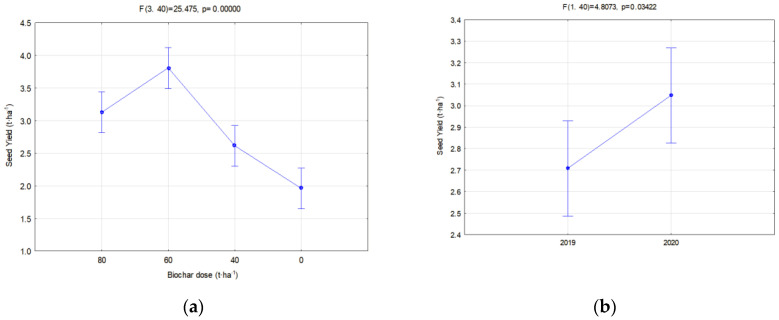
Average yield in relation to the applied biochar dose (**a**) in particular year of research (**b**).

The obtained yields are a good reflection of the hydrological relations on cultivated soils. When analyzing the soya yield, significant differences were found between the facilities where different biochar doses were applied. The highest yields were obtained in a combination where 60 t·ha^−1^ of biochar was implemented, on average ca. 3.8 t·ha^−1^ (Figure 21a). A dose of 80 t·ha^−1^ caused a reduction in the yield. Similarly, Baigorri et al. [7] noticed an unfavorable impact of a dose higher than 5% on the yield of dry mass of wheat. No significant differences in the soya crop yield between the facilities with the various biochars used in the experiment were observed. On the other hand, differences in the years of research occurred. The year 2020 favored soya cultivation (Figure 21b), which was related to the course of atmospheric conditions. Soya is characterized by high water demands in the blooming period, which takes place in June. Low precipitation and high temperatures in June in 2019 (Figure 2 and Figure 3) might have caused the lower soya yield in this year.

**Figure 22:**
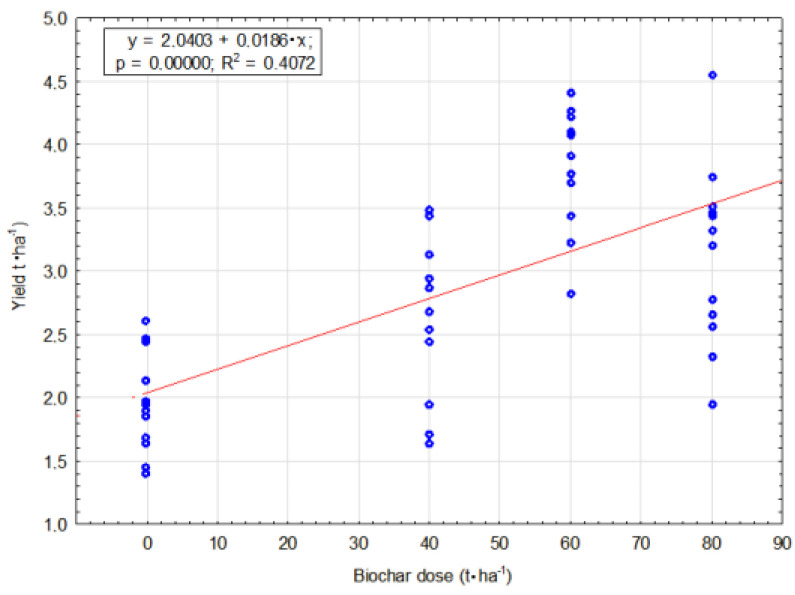
Soya yield relation to applied biochar dose.

The correlation between the biochar dose and soya yield was calculated. Where r was significant (*p* < 0.05) a regression model was determined (Figure 22).

## 4. Discussion

Biochar application to the soils influenced the variability of the majority of their water and physical properties.

### 4.1. Biochar Impact on Soil Physical Properties 

The dry soil bulk density (SBD) depends mainly on its granulation, and only to a small degree on other properties. Investigation of the soil bulk density shows that almost constant results for a particular soil type are obtained (Table 4, Figure 14b). Addition of biochar reduces the levels of the soil bulk density which has been confirmed in numerous studies [39,40,41]. The SBD reduction in soils with biochar addition was strictly related to a the biochar dose and the time after its application (Table 4, Figure 14). Similarly, Obia et al. [40] and Głąb et al. [42] noticed that BC doses play the main role in SBD reduction. 

In the second year of research, the increases in density, in the fallow soils and in those covered with soya were 6.96 and 3.26%, respectively, regardless of the applied dose. This is a normal phenomenon, since natural soil sedimentation takes place and the packing of aggregates in a volume unit increases. However, it is contrary to the research of Xiao et al. [14], who after the second year of research did not observe such changes.

Visible differences between the fallow soils and the soils covered with soya following the application of BC2 should be attributed to mechanical treatments of the soils and the deformation of soil grains as a result of the activity that was carried out. 

The reduction in SBD level was related to the increase in soil porosity. The increase in general porosity and drainage porosity of soils under the influence of biochar introduced to soil has been observed in many previous studies [15,41,43]. The porosity depends mainly on the biochar addition dose. Similar observations were made by Głąb et. al. [42]. In the presented research, a very high SBD and TP and DP correlation was observed. The Pearson coefficient of correlation for these relations, both on fallow soils and those sown with soya, was <0.9 (Table 5 and Table 6). 

It was noticed that, in soils where soya cultivation was carried out, BC2 increased the general porosity of the soils to a higher degree in comparison to those with BC1. Nevertheless, significant differences at the highest applied doses of 60 and 80 t·ha^−1^ were observed (Figure 17b). Moreover, the soils where BC2 was applied and soya was cultivated, showed higher SBD, TP, and DP parameters in comparison to fallow lands (Table 3 and Table 4). Additionally, higher general and drainage porosities after BC2’s application on soils with soya was seen. At the same time, as shown in Table 2, the biochar from forest waste (BC2) was less porous, its total surface area was lower, and its density was higher in comparison to BC1 (ca. 7%). Since the observed phenomena concern only soils where soya was cultivated, it seems that these observations should be related directly to this species. As is commonly known, soya lives in symbiosis with rhizobia. The bacteria responsible for formation of rhizobia need energy for development, the source of which is carbon. BC2 had a greater abundance of carbon, and had a wide relation of C/N (Table 1). Although we have no final explanation of the presented observation, we hypothesise that it is caused by changes in soil packing, which may be related to the intensive formation of rhizobia in the soya root system.

A significant reduction in the soil porosity of fallow soils in the second year of research was noted (Figure 16b,c) in soils where 40 t·ha^−1^ and 60 t·ha^−1^ of biochar was introduced, the general porosity reduced by 14.38 and 13.46%, respectively. 

Similar observations were made by Ni, et al. [44]. In this case, in soils where biochar was introduced in the amount of 10%, within 6–24 months a reduction, of on average 6.8%, was observed in comparison to the biochar application year.

### 4.2. Impact of Biochar on Soil Retention Capabilities

Biochar is commonly suggested as a soil addition, to improve its retention properties which favors plant production. However, as Razzaghi et al. [3] noted in their meta-analysis, non-uniformity between the experiments concerning biochar properties, experimental conditions, and soil properties might cause difficulties in the comparison of results from various studies and providing general conclusions.

From the point of view of cultivations performed, the water content for plants is the most important. In conditions where underground water is located deep and the root system has no contact with capillary infiltration, the water content controls the plant’s vegetation possibility in the periods between precipitation [45]. The analysis of the results of the AWC levels that was carried out after application of BC1, shows the increase in the AWC levels in comparison to the zero test. However, no significant differences between the applied biochar doses of 40, 60, and 80 t·ha^−1^ were noticed. Observations from the BC1 experiment after 12 months prove a reduction in the AWC level, by an average of 7.47%, in comparison to the levels obtained in the biochar application year, and a reduction in FC by an average of ca. 4.15%. Liu et al. [15] pay attention to the fact that biochar’s impact on the water retention of soil may change through time. Ni, et al. [44], when investigating water retention and Cynodon dactylon grass growth with the presence of biochar in soil at the level of 10% for two years, within 6 to 24 months noticed a reduction in the FC and PWP values, by 6% and 8%, respectively. However, it was a considerably lower reduction than on the control soil. Similar observations were also made with the BC2 application.

In the case of BC2, an increase in the AWC level was observed when the biochar dose was increased. Wang et al. [46] showed that high doses (≥10 t·ha^−1^) of biochar, with a large volume of pores and large particle size (≥1 mm), may improve water retention in a thick-grained soil with a limited capability for water storage. Similar observations were obtained by de Melo Carvalho et al. [46] when performing tests on a sandy loam soil with the addition of biochar obtained at a temperature of ∼450 °C from eucalyptus wood in varied doses (0, 8, 16, and 32 Mg·ha^−1^). The study showed an increase in the water available for rice in the upper soil layer (5–10 cm) proportional to the biochar amount, by ca. 0.8% Mg·ha^−1^. However, when comparing the results after BC2 application in considerably higher doses and the abovementioned studies, such a high increase in the amount of water available for the plants was not obtained. The introduction of 40 t·ha^−1^ BC2 to soil resulted in an increase in AWC by an average of 0.22% per ton of the applied biochar, whereas at 60 t·ha^−1^ by an average of 0.29% per ton of biochar, and a dose of 80 t·ha^−1^ caused an average increase of 0.41% per ton of BC2. As was shown in Table 5 and Table 6, the impact of biochar on the AWC was related to the physical properties of the soil, a reduction in SBD, an increase in TP, and the applied dose.

In the second year of research on the soil where BC2 was introduced, a reduction in the AWC, FC, and PPW levels, on average by 16.02%, 10.98%, 2.17%, respectively, in comparison to the previous year was observed. In comparison to BC1, it was a higher reduction in AWC, by 8.55%, and FC by 6.83%. The obtained results may point to the congestion of pores of the biochar introduced to the soil with a varied organic-mineral substance. Wang et al. [46] paid attention to such a phenomenon. With time of use on the site, the biochar particle size may change similarly to the natural soil’s, as a result of physical factors, e.g., cyclic freezing/defreezing [47], or anthropogenic factors related to the manner of soil usage. Soil deformation as a result of crossings of agricultural machines may take place.

The internal porosity of biochar may be reduced by sorption of mineral components [48] and adsorption [7]. A study by Sadowska et al. [4], indicates that the use of biochar at doses of 15 and 45 t·ha^−1^ in coarse soil contributed to an increase in nitrogen utilization by plants after nitrogen fertilization at doses of 100 and 125 kg N·ha^−1^.

BC1 and BC2 addition resulted in a similar effect on the AWC of soil with soya, but slightly different effects in relation to the applied raw material was observed. A similar phenomenon was observed by Obia et al. [40]. The addition of biochar produced from corn cobs had the strongest impact on the hydraulic properties of soil in comparison to biochar from rice husks. A rice husk had no effect on the AWC [40]. The majority of the cited authors did not observe PWP changes in the analyzed soils with biochar. It is assumed that water maintained at the constant point of wilting, and outside it, is maintained under too high a pressure for plants to use it.

In the presented study on fallow soils, biochar addition in the amount of 60 and 40 t·ha^−1^ reduced the PWP level, whereas, in soils with soya, PWP was considerably changed by the biochar type, applied dose, and year of research. On the other hand, Razzaghi et al. [3] showed that, under the biochar application, the coefficients of the permanent wilting point increased considerably for thick-grained soils, by about 47% on average, for medium-grained soils, by about 9%, and in the case of fine soils, the permanent wilting point reduced slightly, by about 5%.

## 5. Conclusions

The results obtained in this experiment show that the use of two biochars from different organic materials significantly improved the physical and hydrological properties of both fallow soils and those where soya was cultivated. The improvement of the soils’ properties is interpreted as an increase in the total porosities and a decrease in the bulk densities of the soils, which increased the water field capacities, plant-available water capacities, and drainage capacities equal to the areal pore volume. 

The number of changes was dependent on the origin of the biochar, the implementation of the biochar, and the applied dose. 

Sunflower husk-derived biochar was found to be more stable and longer-lasting to maintain the resulting soil amendments, considering the range and stability of the changes in soil hydrophysical properties after biochar addition under different treatments.

The type of use of the soils did not affect the hydrological properties of the soils when the same type of biochar was applied.

The presented results will serve as a starting point to continue investigations on the sustainability of the effects of applied biochar.

## Figures and Tables

**Table 1:** Parameters of the feedstock and the produced biochar.

	Moisture (%)	VM * (%)	Ash (%)	C (%)	H (%)	N (%)	HHV * (MJ·kg^−1^)	LHV * (MJ·kg^−1^)
Pellet (sunflower husk)	10.5	66.8	1.8	44.1	5.6	0.6	17.3	16.0
Biochar BC1 (sunflower husk)	13.2	13.8	4.4	74.0	3.6	0.8	27.1	25.8
Pellet (leafy trees)	7.6	76.9	0.7	46.6	5.9	0.2	18.7	17.4
Biochar BC2 (leafy trees)	12.2	8.6	1.8	83.4	3.2	0.3	28.8	27.5

* VM refers to volatile matter content, while HHV and LHV denote higher (gross) and lower (net) heating values, respectively.

**Table 2:** Physical parameters of the biochars.

Components	Bulk Density (g·cm^−3^)	Porosity (%)	Total Surface Area (m^2^·cm^−1^)
Biochar BC1 (sunflower husk)	0.43	18.3	5.8
Biochar BC2 (leafy trees)	0.46	7.9	2.9

**Table 3:** Average values of water and soil constants and selected physical properties of the investigated soils in relation to the dose and type of biochar, and the manner of soil management for the period 2019–2020.

Type of Biochar	Component	Type of Soil Cultivation	Biochar Dose (t·ha^−1^)			
			80	60	40	0
BC1	SBD * (Mg·dm^−3^)	Fallow	1.226	1.312	1.334	1.523
		Soya	1.165	1.304	1.321	1.443
	TP (cm^3^·cm^−3^)	Fallow	0.509	0.475	0.467	0.391
		Soya	0.534	0.479	0.472	0.423
	DP (cm^3^·cm^−3^)	Fallow	0.250	0.205	0.206	0.146
		Soya	0.264	0.214	0.208	0.179
	FC (cm^3^·cm^−3^)	Fallow	0.260	0.270	0.261	0.245
		Soya	0.270	0.264	0.264	0.244
	PWP (cm^3^·cm^−3^)	Fallow	0.094	0.091	0.088	0.092
		Soya	0.095	0.088	0.091	0.096
	AWC (cm^3^·cm^−3^)	Fallow	0.165	0.179	0.173	0.153
		Soya	0.175	0.176	0.173	0.148
BC2	SBD (Mg·dm^−3^)	Fallow	1.252	1.294	1.365	1.515
		Soya	1.092	1.234	1.340	1.454
	TP (cm^3^·cm^−3^)	Fallow	0.509	0.492	0.464	0.394
		Soya	0.572	0.516	0.474	0.419
	DP (cm^3^·cm^−3^)	Fallow	0.232	0.229	0.208	0.152
		Soya	0.280	0.262	0.232	0.181
	FC (cm^3^·cm^−3^)	Fallow	0.277	0.262	0.256	0.242
		Soya	0.292	0.254	0.242	0.237
	PWP (cm^3^·cm^−3^)	Fallow	0.092	0.088	0.092	0.093
		Soya	0.091	0.087	0.090	0.096
	AWC (cm^3^·cm^−3^)	Fallow	0.185	0.174	0.164	0.149
		Soya	0.200	0.167	0.152	0.141

* SBD—soil bulk density, TP—total porosity, DP—drainage porosity, FC—field capacity, PWP—permanent wilting point, AWC—available water capacity.

**Table 4:** Results of the statistical analysis of the impact of the dose and type of biochar and the manner of cultivation on the water and soil constants and selected physical properties of soils as average values for 2019–2020.

Type of Biochar	Component		SBD	TP	DP	FC	PWP	AWC
BC1	Biochar dose (t·ha^−1^)	80	1.196 a	0.522 c	0.257 c	0.265 b	0.095 b	0.170 b
		60	1.308 b	0.477 b	0.210 b	0.267 b	0.090 a	0.178 b
		40	1.327 b	0.469 b	0.207 b	0.262 b	0.089 a	0.173 b
		0	1.483 c	0.407 a	0.162 a	0.244 a	0.094 b	0.150 a
	Cultivation method	Fallow	1.349 a	0.461 a	0.202 a	0.259 a	0.091 a	0.168 a
		Soya	1.308 a	0.477 a	0.216 a	0.260 a	0.092 a	0.168 a
	Year	2019	1.288 a	0.485 b	0.220 b	0.265 b	0.091 a	0.174 b
		2020	1.369 b	0.452 a	0.198 a	0.254 a	0.093 a	0.161 a
	*p*-value	BC1 dose	<0.001	<0.001	<0.001	0.002	<0.001	<0.001
		cultivation method	0.057	0.057	0.097	0.745	0.262	0.262
		Year	<0.001	<0.001	0.020	0.011	0.052	0.003
		BC1 dose and cultivation method	0.529	0.529	0.616	0.545	0.078	0.078
		BC1 dose and year	0.108	0.107	0.012	0.069	<0.001	<0.001
		cultivation method and year	0.051	0.201	0.123	0.463	<0.001	0.030
BC2	Biochar dose (t·ha^−1^)	80	1.172 a	0.540 d	0.256 c	0.284 b	0.092 b	0.193 c
		60	1.264 b	0.504 c	0.246 bc	0.258 a	0.088 a	0.170 bc
		40	1.353 c	0.469 b	0.220 b	0.249 a	0.091 b	0.158 ab
		0	1.484 d	0.406 a	0.167 a	0.240 a	0.094 c	0.145 a
	Cultivation method	Fallow	1.280 a	0.465 a	0.205 a	0.259 a	0.091 a	0.168 a
		Soya	1.356 b	0.495 b	0.239 b	0.256 a	0.091 a	0.165 a
	Year	2019	1.293 a	0.497 b	0.224 a	0.273 b	0.092 b	0.181 b
		2020	1.344 b	0.462 a	0.220 a	0.243 a	0.090 a	0.152 a
	*p*-value	BC2 dose	<0.001	<0.001	<0.001	<0.001	<0.001	<0.001
		cultivation method	<0.001	<0.001	<0.001	0.644	0.695	0.621
		Year	<0.001	<0.001	0.585	<0.001	0.004	<0.001
		BC2 dose and cultivation method	0.008	0.009	0.729	0.373	0.075	0.398
		BC2 dose and year	0.795	0.252	0.720	0.692	<0.001	0.178
		cultivation method and year	0.103	0.089	0.002	<0.011	<0.001	0.004

a, b, c, d—homogenous groups according to Tukey’s test *p* ≤ 0.05.

**Table 5:** Correlation coefficients between the selected physical properties of investigated soils, BC1 biochar dose and its type, and water and soil constants of fallow soils.

Component	FC	PWP	AWC	SBD	TP	DP	Biochar Dose	Type of Biochar
FC	1.000	0.139	0.936 *	−0.548 *	0.561 *	0.262	0.507 *	0.007
PWP	0.139	1.000	−0.217	−0.120	0.107	0.070	−0.014	−0.021
AWC	0.936 *	−0.217	1.000	−0.500 *	0.517 *	0.236	0.507 *	0.013
SBD	−0.548 *	−0.120	−0.500 *	1.000	−0.992 *	−0.941 *	−0.785 *	0.030
TP	0.561 *	0.107	0.517 *	−0.992 *	1.000	0.946 *	0.776 *	0.037
DP	0.262	0.070	0.236	−0.941 *	0.946 *	1.000	0.706 *	0.041
Biochar dose	0.507 *	−0.014	0.507 *	−0.785 *	0.776 *	0.706 *	1.000	0.000
Type of biochar	0.007	−0.021	0.013	0.030	0.037	0.041	0.000	1.000

* Significant relation *p* < 0.05.

**Table 6:** Correlation coefficients between the selected physical properties of investigated soils, BC2 biochar dose and its type, and water and soil constants of soils with soya.

Component	FC	PWP	AWC	SBD	TP	DP	Biochar Dose	Type of Biochar
FC	1.000	−0.347 *	0.982 *	−0.530 *	0.566 *	−0.017	0.444 *	−0.062
PWP	−0.347 *	1.000	−0.518 *	0.220	−0.237	−0.044	−0.233	−0.082
AWC	0.982 *	−0.518 *	1.000	−0.527 *	0.564 *	−0.008	0.452 *	−0.042
SBD	−0.530 *	0.220	−0.527 *	1.000	−0.991 *	−0.829 *	−0.869 *	−0.109
TP	0.566 *	−0.237	0.564 *	−0.991 *	1.000	0.814 *	0.861 *	0.169
DP	−0.017	−0.044	−0.008	−0.829 *	0.814 *	1.000	0.732 *	0.249
Biochar dose	0.444 *	−0.233	0.452 *	−0.869 *	0.861 *	0.732 *	1.000	0.000
Type of biochar	−0.062	−0.082	−0.042	−0.109	0.169	0.249	0.000	1.000

* Significant relation *p* < 0.05.

## Data Availability

Not applicable.

## Abbreviations

AWC: available water capacity, BC1: biochar from sunflower husk, BC2: biochar of leafy trees, DP: drainage porosity; FC: field capacity, HHV: Higher heating value, LHV: Lower Heating Value, PWP: permanent wilting point, TP: total porosity, VM: Volatile matter.
